# Treatment response of advanced HNSCC towards immune checkpoint inhibition is associated with an activated effector memory T cell phenotype

**DOI:** 10.3389/fonc.2024.1333640

**Published:** 2024-03-07

**Authors:** Max Schumacher, Sina Beer, Emmanuelle Moraes Ribeiro, Fulya Korkmaz, Hildegard Keppeler, Rahel Fitzel, Estelle Erkner, Pia Radszuweit, Claudia Lengerke, Corina Schneidawind, Sebastian Hoefert, Paul Stefan Mauz, Dominik Schneidawind

**Affiliations:** ^1^ Department of Hematology, Oncology, Clinical Immunology and Rheumatology, University Hospital Tübingen, Tübingen, Germany; ^2^ Department of Medical Oncology and Hematology, University Hospital Zürich, Zürich, Switzerland; ^3^ Department of Oral and Maxillofacial Surgery, University Hospital Tübingen, Tübingen, Germany; ^4^ Head and Neck Cancer Center, Comprehensive Cancer Center Tübingen-Stuttgart, University Hospital Tübingen, Tübingen, Germany; ^5^ Department of Otolaryngology, University Hospital Tübingen, Tübingen, Germany

**Keywords:** PD-1, HNSCC, immune checkpoint inhibition, peripheral T cells, predictive biomarker, activation marker

## Abstract

Locally advanced or metastatic head and neck squamous cell carcinoma (HNSCC) is associated with a poor prognosis. The introduction of PD-1 inhibitors has led to a significant improvement in survival, but only a subpopulation of patients responds to therapy. Current biomarkers cannot reliably identify these patients. The identification of biomarkers for the prediction and monitoring of immunotherapy is therefore of great importance. In this study, we characterized lymphocyte subsets in the peripheral blood of HNSCC patients under PD-1 inhibition. Patients with primary response (n=11) to PD-1 inhibition showed an increase of the CD3+ effector memory (CD3/EM) population and an elevated expression of the activation marker CD69 in CD3+ T cells, particularly in the CD3/EM subpopulation at 3 months when treatment response was assessed. In contrast, patients with primary treatment failure and progressive disease (n=9) despite PD-1 inhibition had lower absolute lymphocyte counts and an increased expression of CTLA-4 in CD3+ T cells at the time of treatment failure compared with baseline, particularly in CD4+ and CD8+ effector memory populations. Our results demonstrate that HNSCC patients’ response to immune checkpoint inhibition shows a distinct immune signature in peripheral blood, which could help identify refractory patients earlier. Furthermore, strategies to overcome primary therapy failure by inducing a beneficial T cell phenotype or adding alternative immune checkpoint inhibitors could improve response rates and survival of HNSCC patients.

## 1 Introduction

Head and neck squamous cell carcinoma (HNSCC) is a collective term for squamous cell carcinomas originating from the lips, oral cavity, pharynx (naso-, oro-, hypopharynx), larynx, nasal cavity or paranasal sinuses (1, 2). Tumors in the head and neck region represent a relevant health burden and are responsible for approximately 4.5% of all annual cancer deaths and thus for about 450,000 individual deaths worldwide each year (1, 3). The 5-year survival rate for all stages and localization averages about 60%, with large differences in survival rates depending on localization and stage (1, 2). Approximately 2/3 of patients present with an advanced stage (2). At least 50% of these patients already have or will develop distant metastases or local recurrence after primary therapy (2). For locally advanced HNSCC, surgical resection with adjuvant radiotherapy or radiochemotherapy or primary radiochemotherapy, depending on location and resectability, are standard of care. In locally uncontrollable or metastasized HNSCC, combined chemotherapy consisting of platinum, 5-fluorouracil and cetuximab was the standard therapy in first line (4). However, in the last decade, studies have shown a significantly prolonged survival of immunotherapy with blockade of PD-1 receptors by nivolumab or pembrolizumab (5, 6). Since KEYNOTE-048, pembrolizumab has also been recommended for first-line therapy. However, only few patients achieve sustained responses (7). Despite some improvement in survival, approximately half of patients are non-responders, and approximately 30% show progressive disease within 12 months after initial response. However, less than 20% show a long-lasting response (7). In patients with malignant melanoma, it has been shown that many of the patients with a response of more than 12 months often show a sustained response. This leads to a plateau in the Kaplan-Maier curve for progression-free survival typical of checkpoint inhibitors (8). Regarding the overall large group of patients who do not benefit from immunotherapy and show therapy-associated immune-related adverse events (IrAE), the identification of patients who will benefit from immunotherapy is of great importance. In clinical practice, mainly PD-L1 expression is used as a biomarker. The PD-L1 expression of the tumor (TPS) or all cells in a tumor biopsy (CPS) correlates with the response, but does not allow a definite prediction (7, 9). In addition, the determination of PD-L1 expression is not standardized; therefore, reported expression levels are not always comparable (10). Furthermore, in many tumors, there is substantial heterogeneity of tumor microenvironment and PD-L1 expression within the tumor and between primaries and metastases (11, 12). Another much studied biomarker is tumor mutational burden (TMB). Studies have shown that high TMB was associated with an increased response rate (13, 14). The predictive power could be increased again by combining PD-L1 positivity and TMB. However, individual prediction remains out of reach (9). Another challenge in immunotherapy is the evaluation of response to therapy. Currently, this can only be done by imaging and is complicated by pseudo-progressions (15). Therefore, treatment failure can only be identified with delay and response cannot always be reliably identified. Due to the problems described above, immunological biomarkers have recently become the subject of increasing attention in current research. In this context, we hypothesized that there are specific differences in the composition of circulating T cell subpopulations, the expression of activation markers and immune checkpoints that correlate with response to immunotherapy. In the present study, we analyzed the immune profile of peripheral blood lymphocytes in HNSCC patients undergoing therapy with immune checkpoint inhibitors. We aimed at identifying a distinct pattern of cellular subsets and activation markers that is associated with response to therapy.

## 2 Material & methods

### 2.1 Research subjects

20 consecutive patients with advanced or recurrent HNSCC receiving immune checkpoint inhibitor therapy at the University Hospital Tübingen were prospectively studied between April 2020 and August 2023 after written informed consent has been obtained. Clinical data were retrieved from respective chart reviews. This study complies with the Declaration of Helsinki and was approved by the Institutional Review Board of the University of Tübingen (approval 779/2019BO2).

### 2.2 Study design

This is a prospective observational single-center study. Peripheral blood mononuclear cells (PBMCs) were obtained at each treatment cycle prior to the administration of the PD-1 inhibitor. Patients underwent cross-sectional imaging after 3 months to assess treatment response. Based on imaging studies, we divided patients into those with treatment response defined as partial response (PR), stable disease (SD) and mixed response (MR) with continuation of treatment and those with treatment resistance defined as progressive disease (PD) or MR with discontinuation of treatment. Flow cytometry was used to phenotype the PBMCs obtained at each cycle. Cell populations in the peripheral blood samples obtained before the first administration of a PD-1 inhibitor (baseline) and at treatment response assessment 3 months after the start of treatment were compared. Comparisons were made between the two groups and within each group at those two time points. For all patients who responded to treatment, we also analyzed samples at 6 weeks, i.e. after two cycles of pembrolizumab or three cycles of nivolumab.

### 2.3 Differential blood count and flow cytometric analysis

Lymphocytes were counted by a validated automated blood cell counter of the accredited Central Laboratory of the Institute for Clinical Chemistry and Pathobiochemistry at the University Hospital Tübingen. PBMCs were isolated by density centrifugation followed by cryopreservation. For this, 15 mL of Pancoll human (PAN-Biotech GmbH, Germany) was layered with phosphate buffered saline (PBS) diluted blood and centrifuged at 800g for 18 minutes. The PBMC-containing interphase was then aspirated and washed twice with PBS. Cells were frozen in freezing medium containing 20% fetal bovine serum and 10% dimethylsulfoxide at -80°C and transferred to liquid nitrogen after 3 days. Staining of PBMCs was performed using antibodies from BioLegend (San Diego, California, USA) or BD Biosciences (Franklin Lakes, New Jersey, USA). The following surface markers were labeled: CD3 (OKT3, PerCP-Cy5.5), CD4 (OKT4, BV785), CD8 (HIT8a, AF700), CD127 (A019D5, PE-Cy7), CD25 (2A3, BB515), CCR7 (G043H7, BV510), CD45RA (HI100, BV711), CD69 (FN50, PE-Cy5), LAG-3 (11C3C65, PE), CTLA-4 (BNI3, BV421), TIM-3 (F38-2E2, PE-Dazzle594). The viability dye eFluor780 (eBioscience, Waltham, Massachusetts, USA) was used to identify vital cells. Flow cytometric measurements were performed using an LSR Fortessa flow cytometer (BD Biosciences) and analyzed using FlowJo 10.8 (BD Life Sciences). The gating strategy is illustrated in **Supplementary Figures 1A–K**.

### 2.4 Statistical analysis

Statistical analyses were performed using GraphPad Prism 9.4.1 (GraphPad Software, Boston, Massachusetts USA). Survival probabilities were calculated using the Kaplan-Meier method. Comparative statistical analyses were performed using Student’s t-test or log-rank test, and *p* < 0.05 was considered statistically significant.

## 3 Results

### 3.1 Patient characteristics

**Figure 1:**
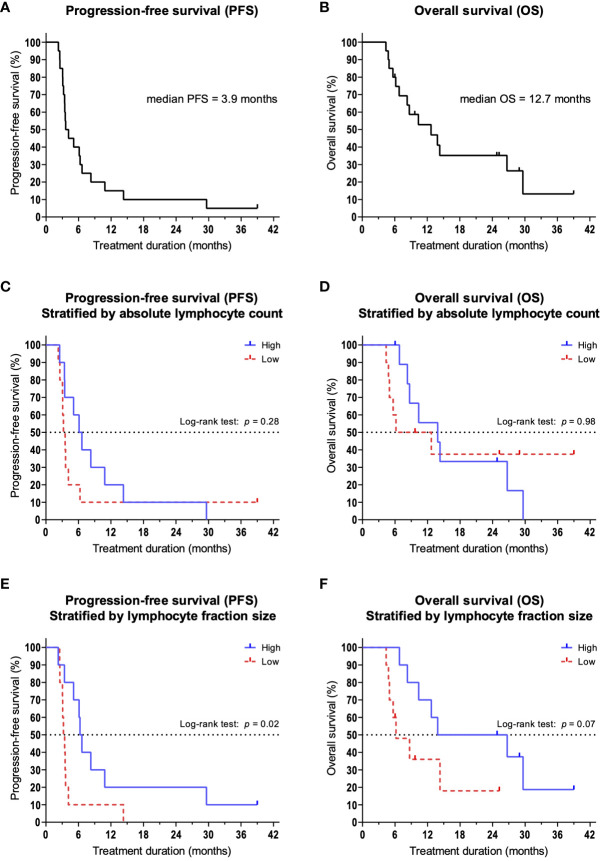
Kaplan-Meier survival curves. Dashes indicate censoring of subjects. **(A)** Overall survival and **(B)** progression-free survival of the overall population. **(C)** PFS and **(D)** OS in patients stratified by the median ALC. **(E)** PFS and **(F)** OS in patients stratified by the median size of lymphocyte fractions.

20 patients were included into the present study. 6 patients were female and 14 patients were male and the median age was 68 years (range 46 – 82). 13 patients received pembrolizumab, 6 patients received nivolumab, and one patient received pembrolizumab plus chemotherapy. All patients had HNSCC, of which 4 were in the oral cavity, 9 were oropharyngeal, 1 hypopharyngeal, 2 laryngeal, 3 in the nasal cavity, and 1 cancer of unknown primary (**Table 1**). Median progression-free survival (PFS) was 3.9 months, and median overall survival (OS) was 12.7 months (**Figures 1A, B**). After 3 months of treatment, 11 patients showed response to therapy (PR, SD or MR with continuation of treatment) and 9 patients showed resistance (PD or MR with discontinuation of treatment). Between patients with therapy response or therapy resistance, there were no significant differences in age (mean: 63.4 vs. 70.9; *p* = 0.15) and CPS (mean: 23.7% vs. 15.3%; *p* = 0.43).

**Table 1:** Characteristics of patients at baseline.

Patient characteristics	Response (n = 11)	Non-response (n = 9)
Age - years		
Median (range)	64 (46 - 81)	71 (51 - 82)
Sex - n (%)		
Male	7 (64)	7 (78)
Female	4 (36)	2 (22)
Primary cancer - n (%)		
Oral cavity	3 (27)	1 (11)
Oropharyngeal	3 (27)	6 (67)
Hypopharyngeal	–	1 (11)
Laryngeal	2 (18)	–
Nasal cavity	3 (27)	–
CUP	–	1 (11)
Stage - n (%)		
Recurrent/Residuum without metastasis	6 (55)	4 (44)
Recurrent/Residuum with metastasis	2 (18)	3 (33)
Solely distant metastases	3 (27)	2 (22)
Systemic immunotherapy - n (%)		
Pembrolizumab	6 (55)	7 (78)
Nivolumab	4 (36)	2 (22)
Chemotherapy and pembrolizumab	1 (9)	0 (0)
PD-L1 CPS - n (%)		
< 1	–	1 (11)
1 - 20	8 (73)	5 (56)
> 20	3 (27)	2 (22)
unknown	–	1 (11)

### 3.2 Reduced lymphocyte counts are associated with resistance to therapy

We hypothesized that lymphocytes, as important effector cells of tumor defense, are increased in patients with therapy response. Therefore, we compared the absolute and relative number of lymphocytes in peripheral blood. We could show that at 3 months, patients responding to therapy had a significantly larger lymphocyte fraction compared to patients with treatment failure (mean: 17.3% vs. 9.4%; *p* = 0.02). At the same time, we found that patients with primary therapy resistance had a significant reduction in relative lymphocyte counts compared to baseline (*p* = 0.04). This also led to a significant reduction in absolute lymphocyte counts (ALC, mean: 1,080/µl vs. 890/µl; *p* = 0.02). Among patients with treatment response, no changes were observed in lymphocyte fractions compared to baseline (*p* = 0.37). Based on the absolute lymphocyte count and the lymphocyte fraction at 3 months, we stratified patients into a high lymphocyte group and a low lymphocyte group using the median. We then analyzed and compared PFS and OS. When categorized by ALC (median = 955/µl), there were no significant differences between the high and low groups in terms of PFS (median 6.4 vs. 3.4 months; *p* = 0.28, **Figure 1C**) and OS (median 13.9 vs. 9.4 months; *p* = 0.98, **Figure 1D**). However, when we categorized patients according to lymphocyte percentage (median = 10,7%), we observed a significantly improved PFS for patients in the high percentage group (median 6.5 vs. 3.3 months; *p* = 0.02, **Figure 1E**). The observed differences in OS were not significant (median 20.3 vs. 6.2 months; *p* = 0.07, **Figure 1F**).

### 3.3 Expansion of the effector memory T cell population is associated with a response to therapy

**Figure 2:**
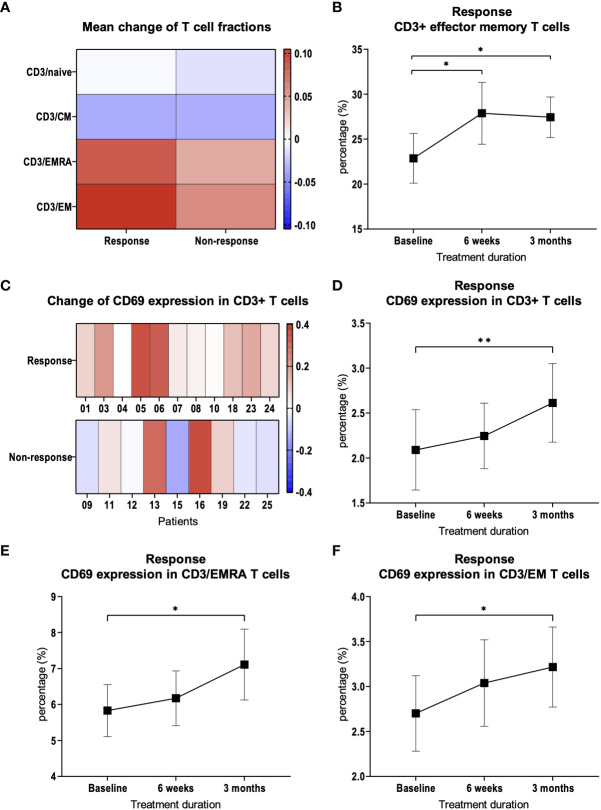
CD3+ T cell subpopulations and CD69 expression. **(A)** Heat map depicting mean of relative changes of CD3+ T cell subpopulations 3 months after therapy initiation compared to baseline. Mean of the relative changes were log10 transformed. **(B)** Percentage of CD3+ EM T cells in patients with therapy response. **(C)** Heat map depicting the relative change of CD69 expression on CD3+ T cells. Each box represents an individual patient and relative changes were log10 transformed. **(D)** Percentage of CD69 expression on CD3+ T cells in patients with therapy response. Percentage of CD69 expression on **(E)** CD3+/EMRA and **(F)** CD3+/EM T cells in patients with therapy response. Bars indicate standard error of the mean. *p<0.05, **p<0.01.

Next, we examined whether patients with a response to therapy showed differences in the subpopulations of T cells in peripheral blood compared to patients with resistance to therapy. For this purpose, we subdivided CD3+ T cells into naïve cells (CD3/Naïve, CD3+/CCR7+/CD45RA+), central memory cells (CD3/CM, CD3+/CCR7+/CD45RA-), effector memory cells (CD3/EM, CD3+/CCR7-/CD45RA-) and terminal differentiated effector cells (CD3/EMRA, CD3+/CCR7-/CD45RA+), as already shown by other research groups (16). In patients with response to therapy, we found a significant increase in the CD3/EM fraction (*p* = 0.03, **Figures 2A, B**) at 3 months compared to baseline. Furthermore, we demonstrated that the increase in CD3/EM fraction among patients with treatment response was already detectable 6 weeks after treatment initiation, i.e. two cycles of pembrolizumab or three cycles of nivolumab (*p* = 0.01, **Figure 2B**). In patients resistant to therapy, there were no significant changes in CD3+ T cell subpopulations over the course of therapy at 3 months. CD3/EM fractions at baseline did not differ between the 2 groups.

### 3.4 Response to treatment is associated with an increased expression of CD69

We suspected that activation of the immune system in response to therapy would lead to the expression of activation markers in addition to changes in CD3+ T cell subpopulations. Therefore, we examined the expression of CD69 during the course of therapy in CD3+ T cells and in the subpopulations. After 3 months, compared to baseline, CD69 expression was significantly increased in patients with response to therapy (*p* = 0.002, **Figures 2C, D**). This observation was most pronounced in CD3/EMRA (*p* = 0.048, **Figure 2E**) and CD3/EM (*p* = 0.03, **Figure 2F**) subpopulations. In patients with treatment failure, there was no significant change in the expression of activation markers in CD3+ T cells compared to baseline (*p* = 0.23).

### 3.5 An increased expression of CTLA-4 in T cells is associated with resistance to therapy

**Figure 3:**
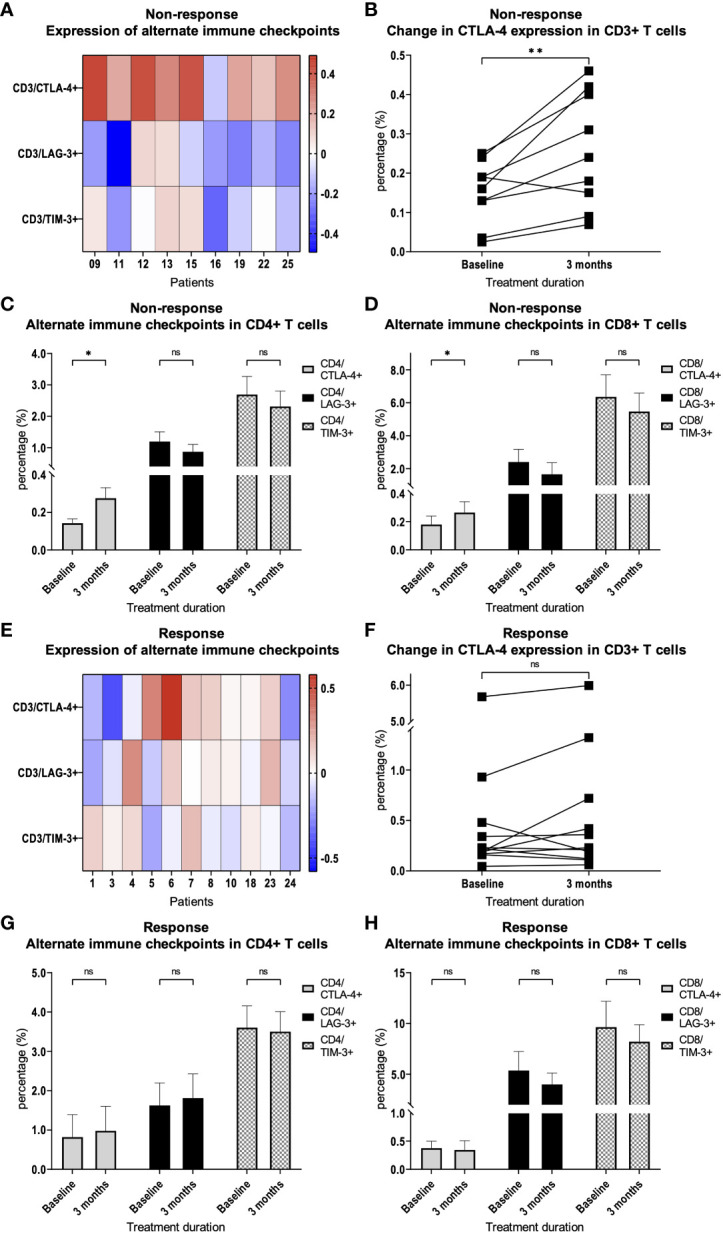
Expression of alternate immune checkpoints on T cells. **(A)** Heat map depicting the relative change of CTLA-4, LAG-3 and TIM-3 expression on CD3+ T cells after 3 months of PD-1 inhibition compared with treatment initiation. Each box represents an individual non-responding patient and relative changes were log10 transformed. **(B)** Percentage of CTLA-4 expression on CD3+ T cells in patients with resistance to therapy. Percentage of CTLA-4, LAG-3 and TIM-3 expression on **(C)** CD4+ and **(D)** CD8+ T cells in patients with therapy resistance. **(E)** Heat map depicting the relative change of CTLA-4, LAG-3 and TIM-3 expression on CD3+ T cells after 3 months of PD-1 inhibition compared with treatment initiation. Each box represents an individual responding patient and relative changes were log10 transformed. **(F)** Percentage of CTLA-4 expression on CD3+ T cells in patients with response to therapy. Percentage of CTLA-4, LAG-3 and TIM-3 expression on **(G)** CD4+ and **(H)** CD8+ T cells in patients with therapy response. Bars indicate standard error of the mean. ns non-significant, *p<0.05, **p<0.01.

We hypothesized that the expression of inhibitory immune checkpoints on T cells may contribute to therapy resistance. We therefore examined whether there are differences or changes in the expression of the immune checkpoints CTLA-4, LAG-3 or TIM-3 on peripheral blood T cells. We were able to demonstrate that there was a steady increase in CTLA-4 expression on CD3+ T cells at response assessment at 3 months compared to baseline in patients with treatment resistance (*p* = 0.009, **Figures 3A, B**). Thereby, the increase in CTLA-4 expression was observed in CD4+ T cells (*p* = 0.01, **Figure 3C**) as well as in CD8+ T cells (*p* = 0.01, **Figure 3D**). In patients who responded to therapy, we did not find a significant increase in CTLA-4 expression on CD3+ T cells compared to baseline (**Figures 3E, F**). This was also true for CD4+ and CD8+ T cell subsets in patients who responded to therapy (**Figures 3G, H**). LAG-3 and TIM-3 expression was not significantly changed in CD3+, as well as CD4+ and CD8+ T cells (**Figures 3A, C-E, G, H**).

**Figure 4:**
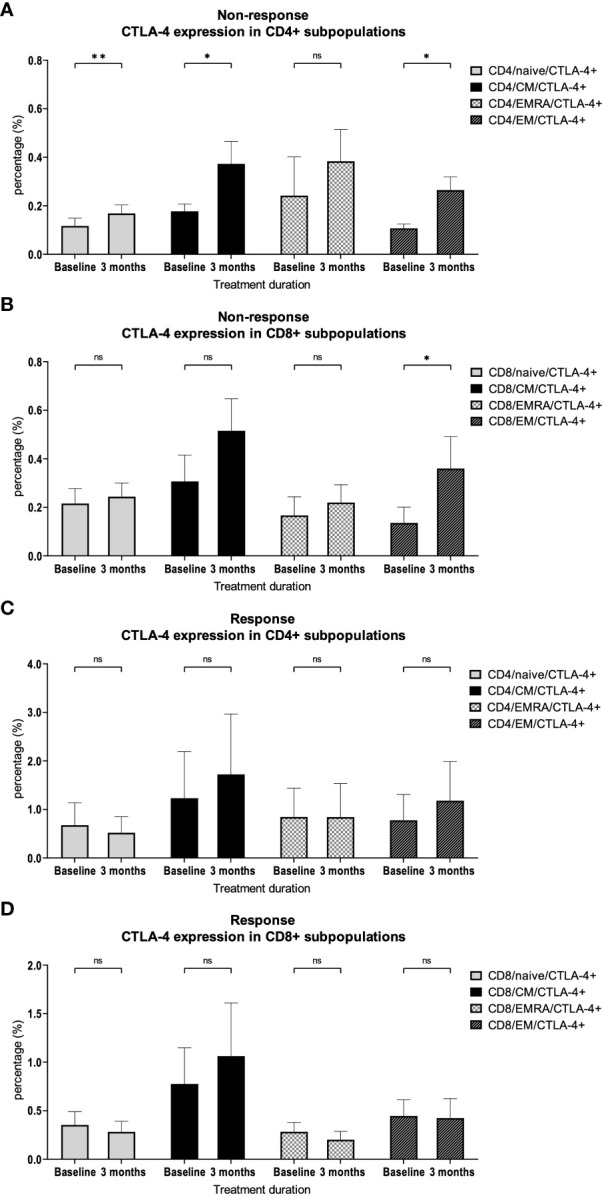
CTLA-4 expression on CD4+ and CD8+ subpopulations. Percentage of CTLA-4 expression on different **(A)** CD4+ and **(B)** CD8+ T cell subsets in patients with therapy resistance. Percentage of CTLA-4 expression on different **(C)** CD4+ and **(D)** CD8+ T cell subsets in patients with therapy response. Bars indicate standard error of the mean. ns non-significant, * *p*<0.05, ** *p*<0.01.

A more detailed analysis of CTLA-4 expression showed a significant increase in almost all CD4+ subpopulations (naïve *p* = 0.009; CM *p* = 0.02; EMRA *p* = 0.39; EM *p* = 0.01) and in the CD8+ EM subpopulation (*p* = 0.049, **Figures 4A, B**) in patients without response to checkpoint inhibition. This effect was not observed in patients who responded to therapy (**Figures 4C, D**).

### 3.6 Regulatory T cell numbers do not correlate with therapy response

Finally, we hypothesized that immunosuppressive regulatory T cells (Tregs, CD3+/CD4+/CD25+/CD127lo) may impair therapy responses to checkpoint inhibitor treatment. However, we did not find significant differences in Treg percentages between patients with therapy response and therapy resistance before therapy (mean: 4.3% vs. 4.9%; *p* = 0.61, **Supplementary Figure 2**) or at response assessment after 3 months of therapy (mean: 4.6% vs. 3.8%; *p* = 0.26, **Supplementary Figure 2**). Furthermore, there were no significant changes in the percentage of Tregs compared to baseline in patients with treatment response (*p* = 0.70) or treatment resistance (*p* = 0.12). However, compared to baseline, we observed a significant increase in LAG-3 expression of Tregs in patients with treatment resistance (*p* = 0.04, **Supplementary Figure 3A**) as well as in patients with treatment response (*p* = 0.049, **Supplementary Figure 3B**).

## 4 Discussion

Various studies link the number and phenotype of peripheral blood immune effector cells with therapy response in solid tumors. For example, in patients with advanced melanoma, it has been shown that a high ALC and a low neutrophil count in peripheral blood 3-6 weeks after initiation of nivolumab treatment are associated with better OS (17). In line with this, it was shown that in advanced non-small-cell lung cancer (NSCLC) a high neutrophil to lymphocyte ratio (NLR) in peripheral blood prior to initiation of therapy with PD-1 inhibitors is associated with a worse PFS and OS (18). In patients with advanced melanoma, an increased ALC in peripheral blood during treatment with ipilimumab was associated with better survival (19). In patients with NSCLC and renal cell carcinoma, it was shown that the peripheral blood of patients with treatment failure had a lower absolute leukocyte count prior to initiation of therapy and a significantly smaller fraction of CD3+ lymphocytes after anti-PD-1 treatment. The study also showed that a larger proportion of CM cells among CD4+ T cells in peripheral blood samples before treatment initiation was associated with therapy response, and an increased or reduced fraction of TIM-3 expressing CD4+ and CD8+ T cells during the course of therapy was associated with therapy failure and therapy response, respectively (20). Fittingly, a high CM/EM T cell ratio in the peripheral blood at baseline was shown to be associated with longer PFS (21). Associations with immune checkpoint blockade were also observed in circulating CD8+ T cells, which are cytotoxic lymphocytes being a central element of tumor defense. Thus, it could be shown that in blood samples taken prior to therapy initiation, the fraction size of CD27+/CD28+ CD8+ EM T cells was positively and CD8+ EMRA T cells were negatively associated with OS (22). The data regarding Tregs is currently contradictory. Consistent with their immunosuppressive function, it has been shown that in patients with advanced melanoma receiving ipilimumab therapy, a decrease in circulating Tregs was associated with improved OS (23), and in patients with NSCLC, a higher percentage of circulating Tregs prior to initiation of PD-1 blockade was associated with a lower response rate (24). However, there have also been studies showing that an increased fraction of Tregs in the peripheral blood prior to ipilimumab therapy was associated with improved OS (25). In light of these conflicting study results, it appears that not only the mere number, but also the immunosuppressive potency of circulating Tregs plays an important role (26).

To test our hypotheses regarding specific differences in T cell subpopulations correlating with response to immunotherapy in HNSCC, we compared PBMCs from HNSCC patients before and during checkpoint therapy. We could show that an increase of CD3/EM T cells during therapy was associated with a response. This highlights the previously shown prognostic relevance of EM T cells (22). The early expansion of CD3/EM cells seems to be important since it was already observable after 6 weeks. Such early changes may represent a possible approach to develop early prediction models for the effectiveness of checkpoint blockade. At the same time, we demonstrated that CD3/EM and CD3/EMRA T cell subpopulations are more activated in response to therapy. This is in line with the systemic activation of the immune system as an important pillar of immunotherapy (27). Interestingly, in mice, CD69 was identified as a relevant factor for the exhaustion of tumor-infiltrating lymphocytes (TILs) (28, 29). Consequently, our data highlight the complex biological function of CD69, which is not fully understood to date, and underscore the need for further research. In addition, we demonstrated that there is increased expression of CTLA-4 in CD3+ T cells in patients with treatment failure. This increased expression was found in diverse CD4+ and CD8+ T cell subpopulations. In CD4+ T cells, we observed an increase in naïve, CM, EM populations which highlights the importance of CD4+ T cells for successful anti-PD-1 immunotherapy (30). In CD8+ T cells, the increased expression was found in the EM population, which also seems to be relevant for successful immunotherapy (22).

Considering the potential clinical relevance of analyzing PBMCs in patients receiving PD-1 blockade, an important question is whether changes in PBMCs also reflect changes in the tumor microenvironment. This probably depends on the clinical situation and distinct cellular subgroups. For instance, correlations between peripheral blood T cell cytotoxicity and TILs have been demonstrated (31), and also neoantigen-specific T cells have been identified in peripheral blood of patients with certain tumors (32). In addition, the expression or blockade of CTLA-4 on circulating T cells also impacts tumor defense (33). In this regard, increased CTLA-4 expression may not only contribute to predict failure of immunotherapy. It could also serve as a potential target to overcome therapy resistance. It has already been shown by other research groups that the increased expression of alternative immune checkpoints is associated with treatment failure (20) and that their blockade potentially overcomes treatment resistance (34). The concept of dual PD-1/CTLA-4 blockade is successfully applied for the treatment of melanoma (8). It has also been studied in HNSCC patients, but failed to show a survival benefit in an unselected patient population (35). However, in a case report, blockade of CTLA-4 with ipilimumab after progression of metastatic HNSCC on nivolumab monotherapy was shown to overcome treatment resistance and produced a long-lasting response (36). Therefore, our study provides a rationale to identify patients who could potentially benefit from additional therapy with ipilimumab and suggests its use in patients with primary PD-1 inhibitor resistance and upregulation of CTLA-4.

In summary, we identified a distinct immune profile in patients with advanced HNSCC that respond to therapy with PD-1 blockade. Given the insufficient predictive power of established biomarkers such as CPS or TMB (7, 9, 13), our data provide important additional insights for the development of more precise models that can be used to monitor immunotherapy or predict responses.

## Data availability statement

The raw data supporting the conclusions of this article will be made available by the authors, without undue reservation.

## Ethics statement

The studies involving humans were approved by Institutional Review Board of the University of Tübingen. The studies were conducted in accordance with the local legislation and institutional requirements. The patients provided their written informed consent to participate in this study.

## Author contributions

MS: Data curation, Formal analysis, Investigation, Methodology, Resources, Visualization, Writing – original draft, Writing – review & editing. SB: Investigation, Writing – review & editing, Formal analysis. ER: Investigation, Methodology, Writing – review & editing. FK: Investigation, Resources, Writing – review & editing. HK: Investigation, Writing – review & editing. RF: Investigation, Writing – review & editing. EE: Investigation, Writing – review & editing. PR: Investigation, Writing – review & editing. CL: Supervision, Writing – review & editing. CS: Supervision, Writing – review & editing. SH: Resources, Writing – review & editing. PM: Resources, Writing – review & editing. DS: Conceptualization, Methodology, Project administration, Resources, Supervision, Writing – original draft, Writing – review & editing.

## Supplementary material

Supplementary Figure 1 Gating strategy. **(A)** Gating of lymphocytes using SSC-A/FSC-A. **(B)** Single cells were gated using FSC-H/FSC-A. **(C)** Living cells were identified by viability dye. Gating of **(D)** CD3+ T cells, **(E)** CD4+ and CD8+ T cells, **(F)** regulatory T cells and **(G)** T cell subpopulations. **(H)** Gating of CD69+ T cells. Gating of the immune checkpoints **(I)** CTLA-4, **(J)** LAG-3 and **(K)** TIM-3.

Supplementary Figure 2 Treg fractions. Percentage of Tregs among CD4+ T cells in responders and non-responders at baseline and after 3 months. Bars indicate standard error of the mean. ns non-significant.

Supplementary Figure 3 Expression of alternate immune checkpoints in Tregs. **(A)** Percentage of CTLA-4, LAG-3 and TIM-3 expression on Tregs in patients with therapy resistance. **(B)** Percentage of CTLA-4, LAG-3 and TIM-3 expression on Tregs in patients with therapy response. Bars indicate standard error of the mean. ns non-significant, * p<0.05.
