# Supplementary figures and images for: Treatment response of advanced HNSCC towards immune checkpoint inhibition is associated with an activated effector memory T cell phenotype
Source: Front Oncol. 2024 Mar 7;14:1333640. doi: 10.3389/fonc.2024.1333640 (PMC10955476; doi:10.3389/fonc.2024.1333640)

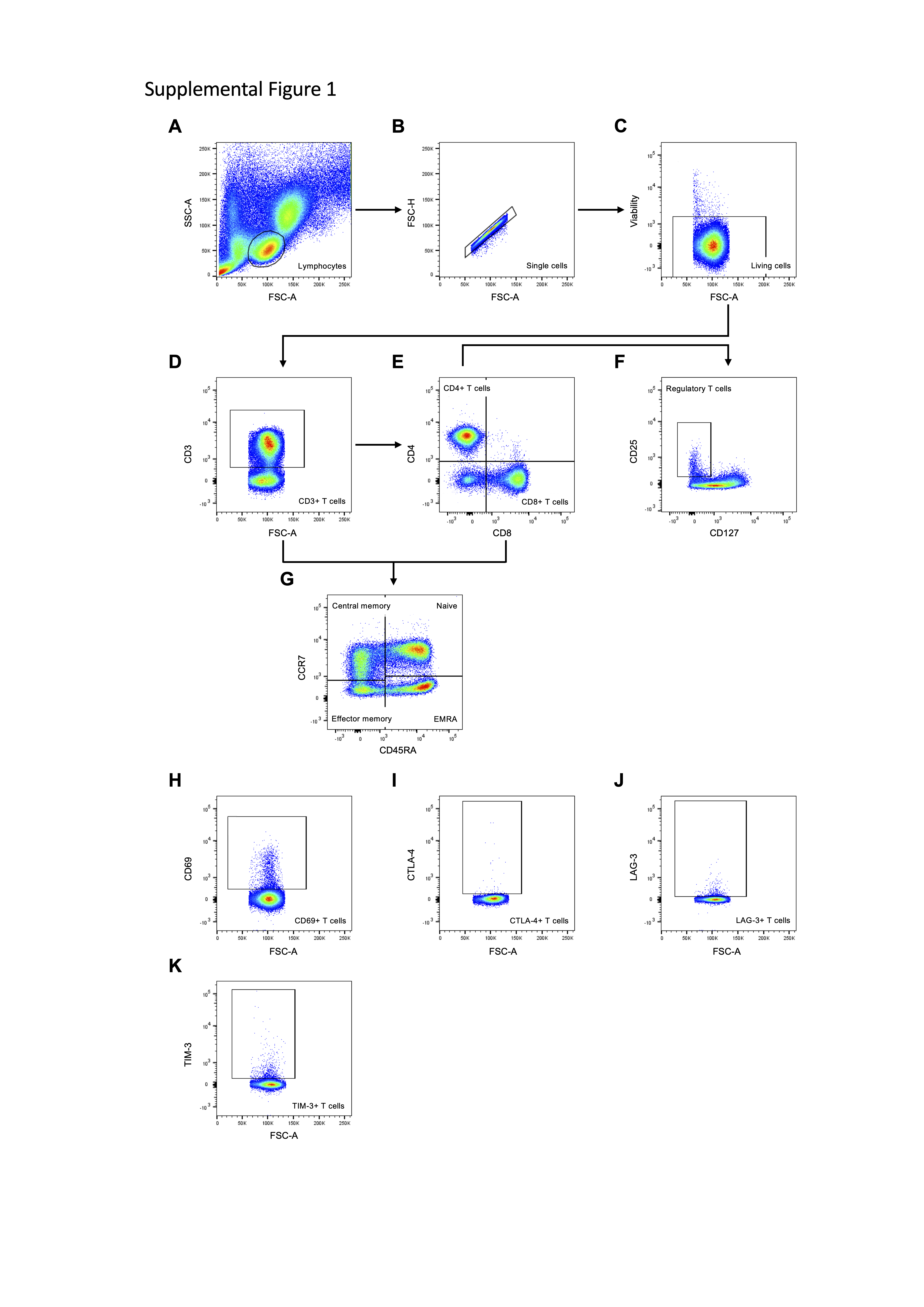

Supplement: Supplementary Figure 1 — Gating strategy. (A) Gating of lymphocytes using SSC-A/FSC-A. (B) Single cells were gated using FSC-H/FSC-A. (C) Living cells were identified by viability dye. Gating of (D) CD3+ T cells, (E) CD4+ and CD8+ T cells, (F) regulatory T cells and (G) T cell subpopulations. (H) Gating of CD69+ T cells. Gating of the immune checkpoints (I) CTLA-4, (J) LAG-3 and (K) TIM-3. [file Image_1.tiff]

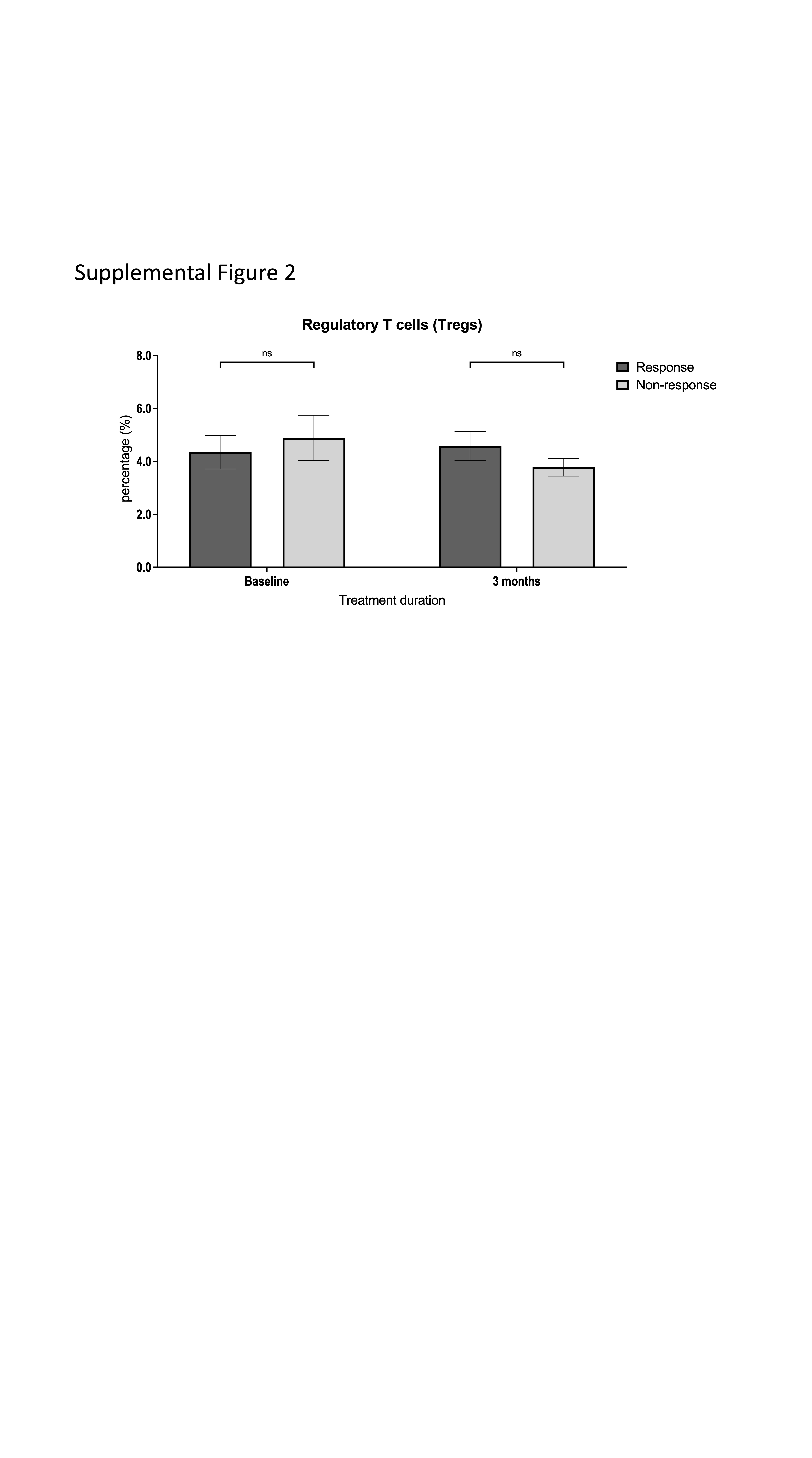

Supplement: Supplementary Figure 2 — Treg fractions. Percentage of Tregs among CD4+ T cells in responders and non-responders at baseline and after 3 months. Bars indicate standard error of the mean. ns non-significant. [file Image_2.tiff]

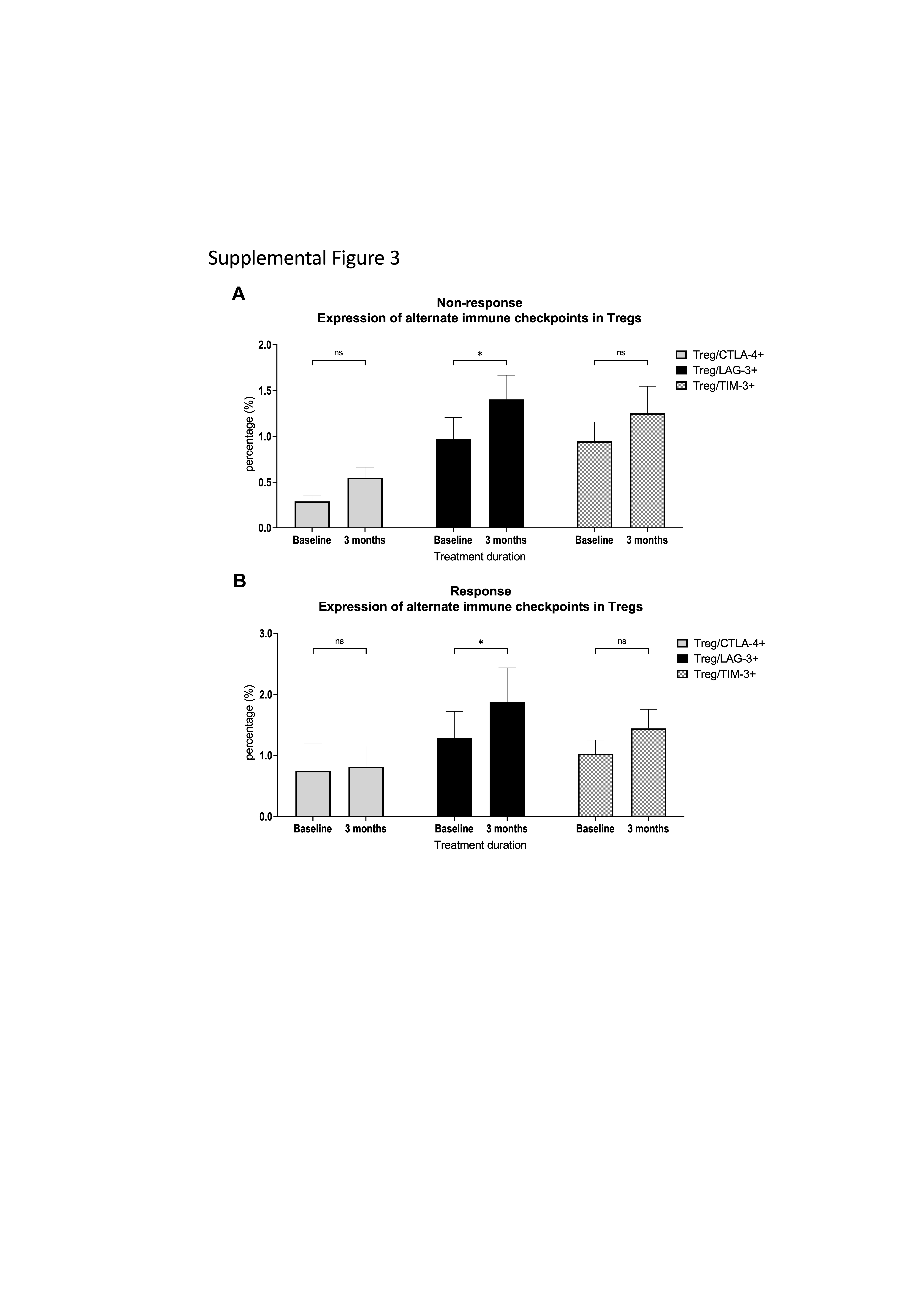

Supplement: Supplementary Figure 3 — Expression of alternate immune checkpoints in Tregs. (A) Percentage of CTLA-4, LAG-3 and TIM-3 expression on Tregs in patients with therapy resistance. (B) Percentage of CTLA-4, LAG-3 and TIM-3 expression on Tregs in patients with therapy response. Bars indicate standard error of the mean. ns non-significant, * p<0.05. [file Image_3.tiff]
